# Co-expression analysis and identification of fecundity-related long non-coding RNAs in sheep ovaries

**DOI:** 10.1038/srep39398

**Published:** 2016-12-16

**Authors:** Xiangyang Miao, Qingmiao Luo, Huijing Zhao, Xiaoyu Qin

**Affiliations:** 1 Institute of Animal Sciences, Chinese Academy of Agricultural Sciences, Beijing, 100193, China

## Abstract

Small Tail Han sheep, including the FecB^B^FecB^B^ (Han BB) and FecB^+^ FecB^+^ (Han++) genotypes, and Dorset sheep exhibit different fecundities. To identify novel long non-coding RNAs (lncRNAs) associated with sheep fecundity to better understand their molecular mechanisms, a genome-wide analysis of mRNAs and lncRNAs from Han BB, Han++ and Dorset sheep was performed. After the identification of differentially expressed mRNAs and lncRNAs, 16 significant modules were explored by using weighted gene coexpression network analysis (WGCNA) followed by functional enrichment analysis of the genes and lncRNAs in significant modules. Among these selected modules, the yellow and brown modules were significantly related to sheep fecundity. lncRNAs (e.g., NR0B1, XLOC_041882, and MYH15) in the yellow module were mainly involved in the TGF-β signalling pathway, and NYAP1 and BCORL1 were significantly associated with the oxytocin signalling pathway, which regulates several genes in the coexpression network of the brown module. Overall, we identified several gene modules associated with sheep fecundity, as well as networks consisting of hub genes and lncRNAs that may contribute to sheep prolificacy by regulating the target mRNAs related to the TGF-β and oxytocin signalling pathways. This study provides an alternative strategy for the identification of potential candidate regulatory lncRNAs.

Sheep are a model species for the study of mechanisms that control ovulation rates, which affect fecundity. In 1980, the Booroola gene (FecB) was described as the first major gene contributing to prolificacy in sheep^1^,^2^. Moreover, three additional fecundity genes have been identified in sheep, including bone morphogenetic protein receptor type IB (BMPRIB)^3^, growth differentiation factor 9 (GDF9)^4^ and bone morphogenetic protein 15 (BMP15)^5^. Sheep are a major component of the global agricultural economy and are a major meat source for human consumption. Numerous different species are found around the world. The Small Tail Han sheep is a native sheep breed of China with hyperprolificacy and has long, strong limbs but a slow growth rate. Dorset sheep are widely bred in the US and have a rapid growth rate. Given their different fecundities, it is valuable to characterize the fecundity genes in these animals. In recent years, the use of transcriptome deep sequencing for the identification of differentially expressed genes has grown^6^,^7^,^8^,^9^. Increasing efforts have focused on revealing the molecular mechanisms contributing to sheep fecundity, such as alterations in DNA, mRNA, methylation, microRNAs (miRNAs) and long non-coding RNAs (lncRNAs)^10^,^11^. Considering the large amount of data generated from RNA-Seq technology, new approaches that efficiently extract meaningful associations from highly multivariate datasets are needed. Weighted gene co-expression network analysis (WGCNA) has been proposed as a solution to systems biology studies to explore the intrinsic organization of transcriptomes^12^. This approach has been successfully utilized to identify the genes, biological processes and pathways involved in cancer and in development in multiple organisms^13^. WGCNA efficiently analyses RNA-Seq datasets by quantifying the correlations between the individual gene pairs and the neighbouring genes. Compared with other techniques based on network analysis, WGCNA transforms gene expression profiles into functional co-expressed gene modules and hub genes that provide insights into the molecular mechanisms associated with development. However, WGCNA has not been applied to studies of sheep fecundity to identify the molecules associated with prolificacy.

In the current study, we identified sheep fecundity-related genes and lncRNAs by comparing sheep with different fecundities. Additionally, we used WGCNA to explore the expression modules and key fecundity-related genes followed by functional annotation for the significant modules. To our knowledge, this is the first time WGCNA has been used to identify the key genes and modules associated with sheep prolificacy.

## Results

### Assembly of the RNA-Seq data

A total of 875,981,773 clean single-end reads with a length of 100 bases were obtained by sequencing all nine libraries. Approximately 7.56 to 11.4 million single-end reads were obtained from each library. Reads were then aligned onto the Ovis aries reference genome using TopHat. Approximately 58% to 74% of the reads were successfully aligned to the Ovis aries reference genome (Table 1).

### Differential gene expression analysis

To study the potential biological functions of the lncRNAs in sheep fecundity, we determined the lncRNA and mRNA expression profiles through RNA-Seq. Compared with Dorset sheep, 1961 and 1849 genes were differentially expressed in Han BB and Han++ sheep, respectively. When compared with Han++ sheep, 1164 genes were differentially expressed in Han BB sheep. The number of differentially expressed lncRNAs is presented in Table 2.

### Construction of the weighted co-expression networks and identification of the fecundity-related modules

**Figure 1:**
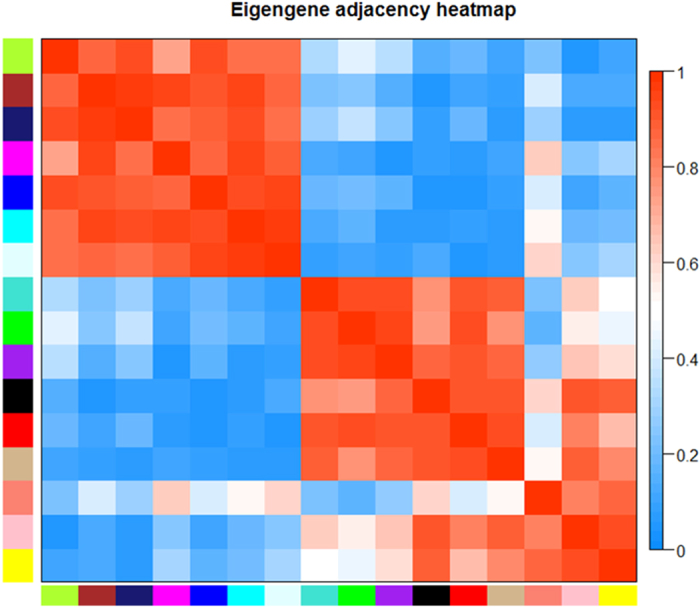
Hierarchical clustering dendrogram of the module eigengenes and a heatmap of the adjacencies using a weighted coexpression network analysis. Blue represents a negative correlation, and red represents a positive correlation.

**Figure 2:**
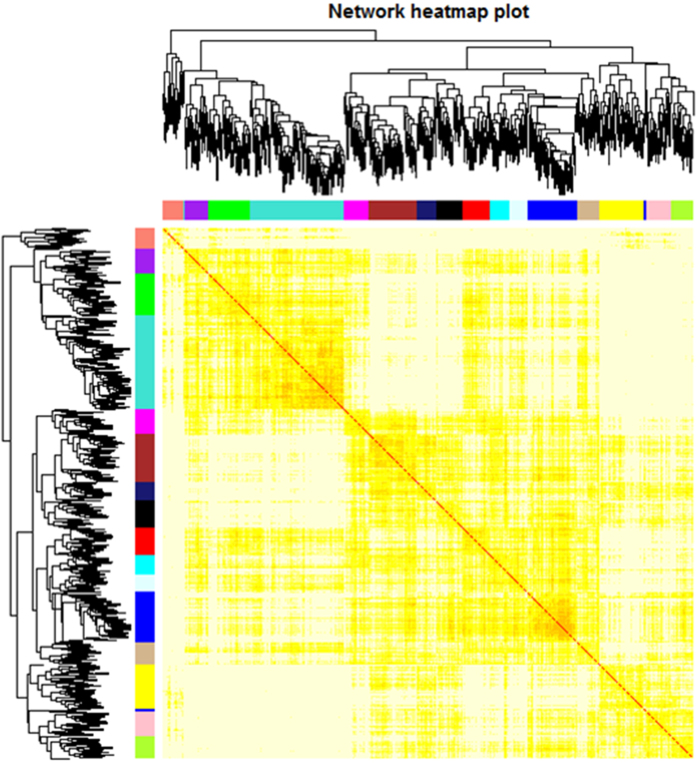
Topological overlap heatmap of the gene coexpression network. Each row and column represent a gene. Light colour indicates low topological overlap, and progressively darker colour indicates increased topological overlap. Darker squares along the diagonal represent modules. The gene dendrogram and module assignment are displayed along the left and top.

To investigate the functional organization of the sheep transcriptome, RNA-Seq of strand-specific libraries from the three groups of sheep was performed. The groups of genes and the lncRNAs exhibiting very similar patterns for each module were then detected using average linkage hierarchical clustering based on the topological overlap calculations. The expression values of the identified genes and lncRNAs of the 9 samples were considered, and a total of 16 modules were screened by extracting the modules containing at least 30 genes and were designed by using different colours in the WGCNA network. A total of 943 genes and lncRNAs were contained in the 16 modules (Table S1). In accordance with the correlation coefficient analysis, the modules that correlated with fecundity (positively or negatively) were clustered (Fig. 1). Furthermore, the genes within the modules exhibited more topological overlap than the genes across the modules in the topological overlap heatmap (Fig. 2).

### Functional annotation of the genes and modules

To examine the extent to which the biological processes and pathways underlying sheep fecundity are shared and differentially regulated, a functional enrichment analysis of the genes of each module was performed, and the Gene Ontology (GO)^14^ terms, including biological process (BP), cellular component (CC) and molecular function (MF), were determined. The results indicated that each module was enriched in different GO terms, although some modules were enriched in the same function. However, most of the enriched functions of the modules were not highly correlated with fecundity (Tables S2,S3,S4,S5,S6,S7,S8,S9,S10).

The significant pathways of the genes in the modules were analysed with the online gene functional classification tool in DAVID. Each module was typically associated with at least one pathway, and more modules were enriched in the same pathways. Notably, the yellow module was involved in the TGF-beta signalling pathway, which is involved in fecundity in various species, including *C. elegans* and Hu sheep^15^,^16^,^17^. The brown module was significantly related to the oxytocin signalling pathway, which is potentially associated with reproductive function^18^. These findings indicate that the pathway significance of the gene expression data of these modules may be very important.

### Identification of the hub genes and lncRNAs in the yellow and brown modules

**Figure 3:**
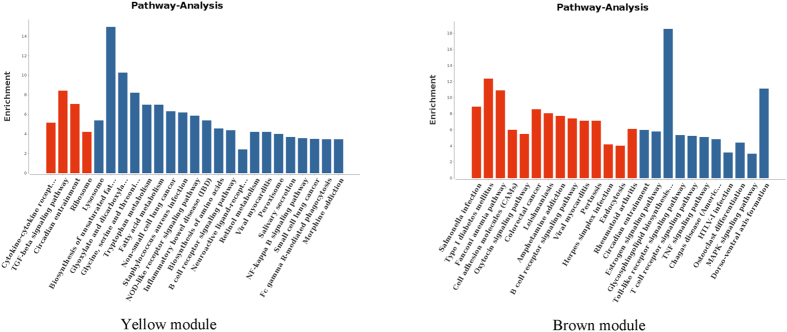
Pathway analyses of all differentially expressed genes in the yellow and brown modules. Red bar refers to the significantly enriched pathway while blue bar refers to the insignificantly enriched ones.

**Figure 4:**
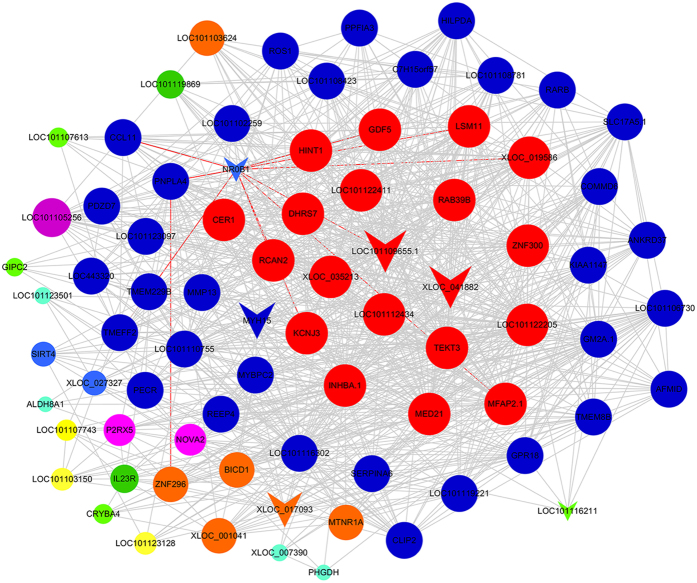
Coexpression network of the differentially expressed genes and lncRNAs in the yellow module. Node colour denotes differential expression levels. Blue represents down-regulation, and red represents up-regulation. Other node colours represent non-differential expression. Node size represents the importance of a node. Edge denotes the interaction strength. Circle nodes represent genes, and inverted triangles denote lncRNAs.

**Figure 5:**
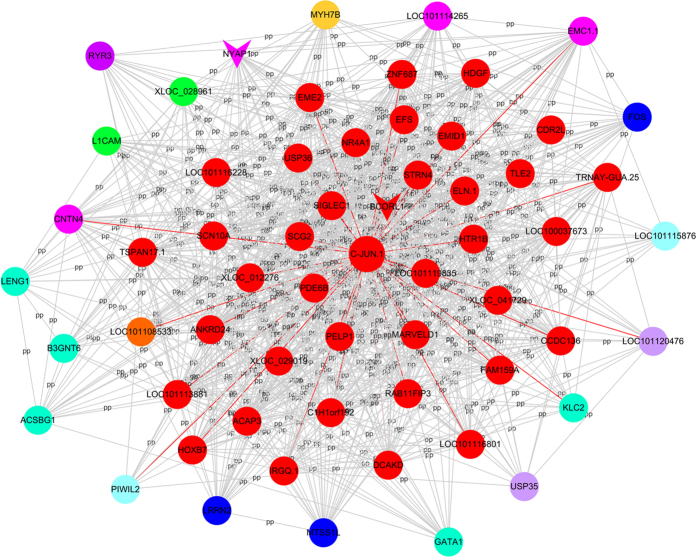
Coexpression network of the differentially expressed genes and lncRNAs in the brown module. Node colour denotes differential expression levels. Blue represents down-regulation, and red represents up-regulation. Other node colours represent non-differential expression. Node size represents the importance of a node. The edge denotes the interaction strength. Circle nodes represent genes, and inverted triangles denote lncRNAs.

The functions and significant pathways of the genes in the yellow module were identified, and the top 5 GO terms are shown in Table 3. We found that these genes were mainly involved in development, proteinase activity and protein complexes. In addition, the differentially expressed genes were significantly related to various pathways, including cytokine-receptor interaction, the TGF-beta signalling pathway, circadian entrainment and ribosomes. The enriched pathways in the yellow and brown modules are presented in Fig. 3. WGCNA of the genes in the yellow module revealed that 6 lncRNAs regulated 73 genes in the co-expressed network (Fig. 4). These 6 lncRNAs consisted of nuclear receptor subfamily 0, group B, member 1 (NR0B1); LOC101109655.1; XLOC_041882; myosin, heavy chain 15 (MYH15); XLOC_017093 and LOC101116211. WGCNA indicated that most genes in the network exhibited a higher K-core, such as potassium inwardly rectifying channel, subfamily J, member 3 (KCNJ3); growth differentiation factor 5 (GDF5) and mediator complex subunit 21 (MED21). Additionally, two key lncRNAs, neuronal tyrosine-phosphorylated phosphoinositide-3-kinase adaptor 1 (NYAP1) and BCL6 corepressor-like 1 (BCORL1), were located at the core of the network for the brown module (Fig. 5). Notably, C-JUN interacted with many differentially expressed mRNAs and was a key gene in the brown module.

### Validation of the RNA-Seq data by real-time PCR

**Figure 6:**
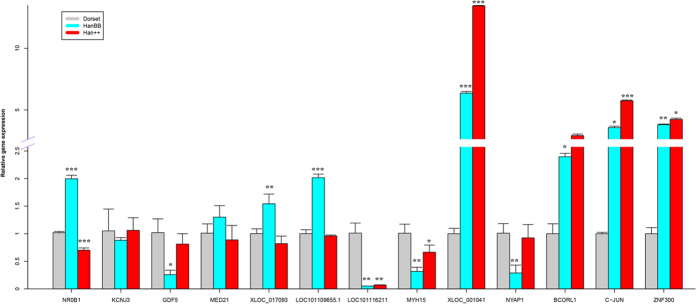
qRT-PCR validation of selected differentially expressed lncRNAs and genes. The relative expression level of each lncRNA and gene was normalized to 18S rRNA.

To validate the RNA-Seq data, the 7 candidate lncRNAs and 5 genes with the highest k-core differences were selected from the network for confirmation by RT-PCR (Table 4, Fig. 6). These confirmations indicated that the data regarding the differential gene and lncRNA expression were reliable. The RT-PCR and RNA-Seq results exhibited clear correlations.

### Target gene prediction of lncRNAs and interaction network construction

**Figure 7:**
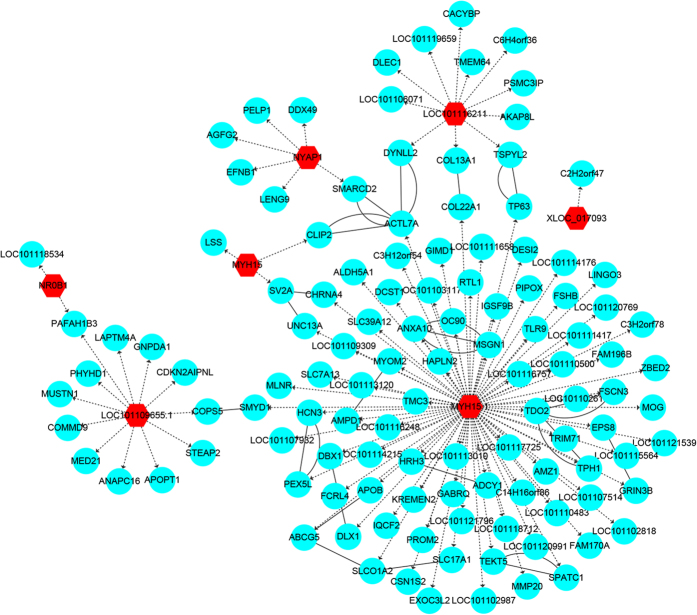
Interaction network for lncRNAs and trans-regulated targets related to reproduction. Red hexagon indicates differentially expressed lncRNA and cyan circle indicates target genes. Dotted arrow represents trans-regulation between lncRNA and target genes. Solid line represents the interaction of target genes.

To explore how lncRNAs might participate in regulating fecundity, we attempted to predict the cis- and trans-regulated target genes of the differentially expressed lncRNAs. There were only 4 lncRNAs that were predicted to have cis-regulated target genes (Table S11). In terms of trans-regulated target genes, most of lncRNAs were co-expressed with more than five coding genes (Table S12). Since most lncRNAs regulated trans-target genes, the lncRNA-mRNA trans-regulated interaction network related to fecundity was constructed (Fig. 7). We found that MYH15 regulated most of mRNAs in the network.

## Discussion

The primary goal of the current study was to identify the genes and lncRNAs associated with sheep fecundity and to examine how these molecules contribute to the molecular mechanisms of sheep prolificacy. Our analyses revealed distinct fecundity-specific co-expression networks and biological functions of these modules. This study provides critical insight into the transcriptional mechanisms underlying different fecundities.

We identified significant differential expression of a number of genes and lncRNAs among different sheep groups. After constructing co-expression networks for differentially expressed mRNAs and lncRNAs of three comparison groups, a total of 16 significant modules were obtained. A functional annotation analysis indicated that the fecundity-associated yellow module of co-expressed genes was enriched for the TGF-beta signaling pathway. Given that the paracrine and autocrine effects of TGF-β on tumour cells and their micro-environment exert both positive and negative effects on tumourigenesis, the TGF-β signaling pathway plays critical roles in tumour suppression and progression^19^,^20^. A previous study has demonstrated that the TGF-β signaling pathway is essential for *Drosophila* oogenesis^17^. Moreover, the members of the TGF-β superfamily, including growth differentiation factor 9 (GDF9) and bone morphogenetic protein 15 (BMP15), are essential for the normal follicular development and function of ovarian cells in sheep and humans^4^,^5^,^21^,^22^. Additionally, the genes in the brown module were significantly enriched in the oxytocin signaling pathway, which is involved in rabbit reproduction^18^. Accordingly, the genes and lncRNAs in the yellow and brown modules were used to construct the co-expression networks.

Six lncRNAs in the yellow module (NR0B1, LOC101109655.1, XLOC_041882, MYH15, XLOC_001041 and LOC101116211) were correlated with sheep fecundity via regulation of the TGF-β signaling pathway. For example, NR0B1 (encoded by the Nr0b1 gene), also known as the dosage-sensitive sex reversal, adrenal hypoplasia congenital critical region on the X-chromosome, gene 1 (Dax1), is an orphan nuclear receptor regulating the expression of steroidogenic enzymes in mice^23^,^24^. Nr0b1 is over-expressed in ovarian tissue but is weakly expressed in the testicular tissue of the protandrous black porgy fish. In addition, Nr0b1 down-regulates nr5a4-mediated cyp19a1a expression in the ovarian follicles in medaka^25^,^26^. Furthermore, Nr0b1 is also crucial for either male or female sex differentiation in various species, such as frogs^27^, mice^28^, pigs^29^ and chickens^30^. Notably, Nr0b1 expression levels may regulate the timing of oocyte development and vitellogenesis for sex change in protandrous black porgy fish^26^. lncRNAs regulate mRNA expression via several mechanisms, such as enhancer RNA (eRNA), competing endogenous RNA (ceRNA) and lncRNA-DNA methyltransferase (DNMT) or lncRNA-transcription factor (TF)^31^. Target gene prediction for differentially expressed lncRNAs showed that most of lncRNAs exerted function in sheep fecundity via trans-regulatory target gene. In this study, NR0B1 regulated more than 10 genes in the yellow module, including growth differentiation factor 5 (GDF5). GDF5 is important for joint formation^32^. In addition, another GDF family member, GDF9, is the first identified TGF-β family member and oocyte-secreted factor associated with fertility in mammals^33^. Considering the relationship between Nr0b1 and GDF5, we hypothesize that Nr0b1 plays a vital role in sheep fecundity by regulating the expression of GDF5 and other genes, which were differentially expressed in the TGF-β signaling pathway. Meanwhile, our previous study showed that a similar regulatory relationship in Han BB compared with Han++. There were only two lncRNAs in the networks, including XLOC_041882, which are connected to two and one differentially expressed mRNAs. Furthermore, we also revealed that lncRNAs and miRNA can form complex regulatory networks and participated in many biological processes. With the presentation of the hypothesis of competing endogenous RNA (ceRNA), our previous findings suggest that lncRNAs could be regulated by miRNAs and thereby favour the expression of repressed mRNA targets. For example, chrx_30776_star and MYH15 interact with each other, indicating that MYH15 may combine with chrx_30776_star through competition with other target mRNAs^34^.

In addition, another lncRNA, XLOC_041882, was correlated with several genes, such as zinc finger protein 300 (ZNF300). Zinc finger protein is one of the most important transcription factors and plays an important role in regulating gene expression^35^. More recently, ZNF300 and its novel splice variant were observed to be highly expressed in human testis, suggesting their essential roles in prolificacy^36^. In addition, a pseudo-gene of the human ZNF300, ZNF300P1, which shares 89% identity with ZNF300, is a long-intergenic non-coding RNA that is frequently methylated in ovarian cancer, indicating a potential role for ZNF300P1 expression in regulating ovarian cancer cell metastasis^37^.

In the co-expression network of the genes in the brown module, NYAP1 and BCORL1 lncRNAs regulated most of the differentially expressed mRNAs. Interestingly, BCORL1 is associated with premature ovarian failure, and its deletion may lead to the insufficient repression of apoptosis, resulting in the atresia of ovarian follicles^38^. The core gene of the network, C-JUN, is a transcription factor that plays key roles in many biological processes, ranging from cell survival to cell death^39^. In addition, C-JUN is selectively expressed in mitotically active, non-differentiating granulose cells of rats, suggesting that it may promote proliferation rather than differentiation of granulose cells^40^. Therefore, the lncRNAs NYAP1 and BCORL1 play key roles in ovary development by regulating the target genes associated with the oxytocin signaling pathway, and their differential expression levels in Han sheep may contribute to the increased fecundity rate.

There was one limitation in our study that we merely provided some indirect experimental evidence to indicate the functional link between lncRNA and its potential target gene, but cannot confirm the defined results limited by the research approaches. Nevertheless, we will demonstrate our predictions of correlation between lncRNA and genes and illustrated the functional roles of these lncRNAs in sheep fecundity in future research.

## Conclusions

Overall, the current study systematically reveals the inherent functional modules that are uniquely activated in the prolificacy of sheep by using a WGCNA approach. Such an approach will facilitate large-scale gene expression studies for the investigation of the molecular mechanisms of sheep fecundity and the identification of the associated lncRNAs contributing to fecundity differences. These results indicated that several lncRNAs may be associated with the fecundity rate of Han sheep by regulating the differential expression of proteins related to the TGF-β and oxytocin signaling pathways. Our findings provide an alternative strategy for the identification of potential candidate lncRNAs and an avenue to guide further efforts to overcome the barriers to sheep prolificacy.

## Materials and Methods

### Ethics statement

All of the procedures involving animals were approved by the animal care and use committee of the Institute of Animal Sciences, Chinese Academy of Agricultural Sciences, where the experiments were conducted. All of the experiments were performed in accordance with the relevant guidelines and regulations set by the Ministry of Agriculture of the People’s Republic of China.

### Animals and tissue samples

A total of 108 adult Han ewes from a fine nucleus herd and 5 adult Dorset ewes aged from 2.5 to 3 years old were bred at the Ao-Te sheep breeding farm in Qingdao (Shandong, China). Blood from the Han ewes was used to identify the FecB mutation of the BMPR1B gene. Han ewes with the BB or ++ genotype served as high-fecundity Han sheep. Dorset ewes with low-fecundity served as the controls. All animals were maintained with food and water *ad libitum* under natural lighting and temperature conditions. All experiments complied with the rules established by the Ministry of Agriculture of the People’s Republic of China.

In total, 3 Dorset sheep (Dorset), 3 Small Tail Han FecB^+^FecB^+^ sheep (Han++) and 3 Small Tail Han FecB^B^FecB^B^ sheep (Han BB) were used for the experiments. All the experimental ewes were treated with fluorogestone acetate vaginal sponges (40 mg; Chronogest, Intervet, Federal District, México) for 10 days. Then, the sheep were intramuscularly injected with pregnant mare’s serum gonadotropin (Ningbo Hormone Co., Ningbo, China) at a dose of 400 IU for synchronized oestrus^41^. All ewes were euthanized between 24 and 36 h after having reached spontaneous oestrus after one cycle. Whole ovary samples with ovulation points were dissected immediately after death, and tissue samples were snap frozen in liquid nitrogen and stored at −80 °C until analysis.

### RNA isolation and sequencing

The total RNA from the ovaries was extracted by using TRIzol reagent (Invitrogen, Carlsbad, CA), and the RNA integrity number (RIN) value of all the samples was greater than 8. RNA was purified with an RNeasy MiniElute Kit according to the manufacturer’s instructions (Qiagen, Valencia, CA). A TruSeq RNA Sample Preparation Kit (Illumina, San Diego, CA) was used for the library construction. The libraries were then assessed with an Agilent Technologies 2100 Bioanalyzer and an Agilent High Sensitivity DNA Kit (Agilent Technology, US). The pooled libraries were sequenced on an Illumina HiSeq2000 (Illumina, San Diego, CA). After sequencing, the indexed adapter sequences were trimmed by using CASAVA software (Illumina).

### Transcriptome assembly and data analysis

RNA-Seq reads from each sample were aligned to the oar 3.1 sheep reference genome with TopHat^42^ using the default settings. Only uniquely mapped reads were used for gene expression analysis. According to the rigorous significance test for the digital gene expression profiling described previously, the DESeq package was used to identify significantly differentially expressed genes (DEGs)^43^. False discovery rate (FDR) was used for the error rate adjustment in multiple significance tests^44^. If fold change >1.5 or <0.667, and FDR < 0.05, the genes and lncRNAs were considered to be differentially expressed.

### Target prediction

Differentially expressed lncRNAs were selected for target prediction via cis- or trans-regulatory effects. For each lncRNA, Pearson correlation of its expression value with that of each mRNA was calculated. For the cis pathway target gene prediction, the genes transcribed within a 20-kb window upstream or downstream of lncRNAs and the Pearson correlation of lncRNA-mRNA more than 0.9 were considered as cis target gene. RNAplex software was then used to select trans-acting target genes with correlation value more than 0.75. Moreover, the interaction network of the differentially expressed lncRNAs and their trans-target genes related with reproduction were constructed based on the String database (http://string-db.org/).

### Gene module construction

To begin analysis, the differentially expressed transcripts and lncRNAs were identified in three control groups. Co-expression network analysis was performed by using the WGCNA R package^45^. To build the co-expression networks for the detected DEGs and the differentially expressed lncRNAs, an unsupervised co-expression relationship was first built according to the adjacency matrix connection strengths by calculating Pearson correlation coefficients for the gene pairs followed by conversion of the correlation matrix into an adjacency matrix. On the basis of the resulting adjacency matrix, topological overlap measures, which consider the correlation of the gene pairs and their shared relationships in the weighted gene network, were calculated. Average linkage hierarchical clustering on the topological overlap was performed to group genes with highly similar co-expression relationships. Modules containing at least 30 genes were assigned using a mixed dynamic tree-cutting algorithm, and module eigengenes were subsequently calculated for each module. Each module was subsequently assigned a colour and a network, and the detected modules were visualized as a hierarchical clustering tree. The node and edge information of each module network were exported as a Cytoscape file by using Cytoscape to visualize and analyse the network modules^46^.

### Functional annotation of the hub genes and modules

GO and Kyoto Encyclopedia of Genes and Genomes (KEGG)^47^ enrichment analysis of the module was performed on the basis of the analyses of the genes in each module. To test the module for enrichment of the genes with significant GO terms and KEGG categories, DAVID was applied^48^. A p-value of less than 0.05 was chosen as the threshold.

### Real-time PCR validation

The total RNA was reverse transcribed using a Qiagen miScript II Reverse Transcription Kit (QIAGEN, Valencia, CA) according to the manufacturer’s instructions. Then, the synthesized cDNA was used as template for real-time PCR reactions. A LightCycler 480 SYBR Green I Master was used to measure mRNA and lncRNA expression levels. Real-time PCR reactions were performed using a Roche LightCycler 480 II system. The internal control genes encoding 18 S rRNA and the comparative Ct method were used to calculate the relative expression levels.

### Statistical Analyses

All of the data are presented as the mean ± SD. The significance of differences among three groups was determined by one-way ANOVA analysis of variance followed by Student’s t-test for quality of variances using SPSS 17.0 (IBM, USA). Differences at p < 0.05 was considered statistically significant.

## Additional Information

**How to cite this article**: Miao, X. *et al*. Co-expression analysis and identification of fecundity-related long non-coding RNAs in sheep ovaries. *Sci. Rep.* **6**, 39398; doi: 10.1038/srep39398 (2016).

**Publisher’s note:** Springer Nature remains neutral with regard to jurisdictional claims in published maps and institutional affiliations.

## Supplementary Material

Supplementary Information

Supplementary Table S2

Supplementary Table S3

Supplementary Table S4

Supplementary Table S5

Supplementary Table S6

Supplementary Table S7

Supplementary Table S8

Supplementary Table S9

Supplementary Table S10

Supplementary Table S11

Supplementary Table S12

## Figures and Tables

**Table 1:** Summary of the reads mapping to the ovary transcriptomes.

	D4	D5	D6	W1	W2	W4	W6	W7	W8
Number of reads	88,473,074	89,641,192	75,615,000	103,878,765	105,666,818	89,679,114	114,102,676	104,406,118	104,519,016
Mapped reads	58,252,020	67,053,070	56,035,422	74,974,042	6,206,6174	64,503,305	82,264,939	76,634,078	61,489,225
Mapping rate (%)	65.8	74.8	74.1	72.2	58.7	71.9	72.1	73.4	58.8
Unique mapped reads	53,328,530	62,718,481	53,377,077	65,796,858	55,305,047	61,683,099	74,596,741	69,903,702	55,674,758
Unique mapped rate (%)	60.3	70.0	70.6	63.3	52.3	68.8	65.4	67.0	53.3

Raw gene expression levels were estimated by measuring the normalized count number for each transcript (number of reads per transcript divided by the total number of mapped reads for each sample).

**Table 2:** Number of differentially expressed long non-coding RNAs (lncRNAs) identified from the three sheep groups.

Comparison groups	Number of differentially expressed lncRNAs	Number of up-regulated lncRNAs	Number of down-regulated lncRNAs
Han BB vs. Dorset	138	62	76
Han BB vs. Han++	106	40	66
Han++ vs. Dorset	138	86	52

**Table 3:** Gene Ontology (GO) enrichment analysis of the genes in the yellow module.

ID	Description	Difgene	P-value
GOBP:0003417	growth plate cartilage development	2	0.000598179
GOBP:0048048	embryonic eye morphogenesis	2	0.000598179
GOBP:0032331	negative regulation of chondrocyte differentiation	2	0.001826261
GOBP:0006541	glutamine metabolic process	2	0.002914391
GOBP:0050850	positive regulation of calcium-mediated signalling	2	0.003416278
GOMF:0036122	BMP binding	2	0.000450326
GOMF:0001968	fibronectin binding	2	0.004202953
GOMF:0004061	arylformamidase activity	1	0.00706204
GOMF:0015136	sialic acid transmembrane transporter activity	1	0.00706204
GOMF:0032428	beta-N-acetylgalactosaminidase activity	1	0.00706204
GOCC:0032982	myosin filament	2	0.002171606
GOCC:0002141	stereocilia ankle link	1	0.006837782
GOCC:0072517	host cell viral assembly compartment	1	0.006837782
GOCC:0043509	activin A complex	1	0.006837782
GOCC:0072536	interleukin-23 receptor complex	1	0.010238828

BP, biological process; MF, molecular function; CC, cellular component; Difgene, differential gene.

**Table 4:** Verification of gene expression changes by qRT-PCR.

Gene	Dorset	HanBB		Han++		Forward primer	Reverse primer	
NR0B1	1.0188 ± 0.0226	1.996 ± 0.0623	***	0.7014 ± 0.0410	***	AACTCAACAGATACTGAGCGAA	GATGAATCTCAGAAGGAAGAGG	
KCNJ3	1.0512 ± 0.3961	0.8816 ± 0.0497		1.0615 ± 0.2276		GTCATTGCTCATTCAAACACC	TTCCATTGTGTTACTCTGCTT	
GDF5	1.0193 ± 0.2503	0.2573 ± 0.0820	*	0.8122 ± 0.1883		CGTAAGTGGGAGAGAACTTGA	CAATAGGTGAAGAGGAAGTCG	
MED21	1.0097 ± 0.1691	1.3000 ± 0.2092		0.8883 ± 0.2619		GTCTCTTTGTTTGCGGAGG	CAGCACTCCAATGGCGTTA	
XLOC_017093	1.0025 ± 0.0866	1.5436 ± 0.1754	**	0.8212 ± 0.1357		TATCACTGGAGGCGTGTC	AAACCACTGCGTGCCTTA	
LOC101109655.1	1.0017 ± 0.0703	2.0147 ± 0.0655	***	0.9594±0.0168		CAGGTACACTTTCAAGGGC	CTCTTTGGAACTATTCCATGC	
LOC101116211	1.0113 ± 0.1822	0.0525 ± 0.0008	**	0.0693 ± 0.0045	**	GCGGGGCACCCTTTGAT	GGTGGAGAAAAGAGGCTGAATAACT	
MYH15	1.0085 ± 0.1647	0.3177 ± 0.0744	**	0.6657 ± 0.1285	*	TCTGTAGTCTGTATGAGGAACG	CTCACCATTCTCACTCCG	
XLOC_001041	1.0000 ± 0.0975	6.3695 ± 0.1302	***	13.4393 ± 0.0299	***	TCCTGGGCACTGACCAAGAG	GAAATGAATGGCAGATCACGAAC	
NYAP1	1.0095 ± 0.1742	0.2894 ± 0.1420	**	0.9249 ± 0.2414		GTGCTGAATAAGGGCTGT	CAGGAGATGCTGGCAAAG	
BCORL1	1.0000 ± 0.1802	2.3958 ± 0.0609		2.9237 ± 0.1192	*	GGATGGTGATGTGGTCTTCAACTT	GAGTCTGGTTGCCACTTGTTCTTAC	p < 0.001***
C-JUN	1.0000 ± 0.0257	3.5769 ± 0.1107	*	5.7455 ± 0.0632	***	AAGATGGAAACGACCTTCTACGAC	GGGTCATGCTCTGCTTCAGAAT	p < 0.05*
ZNF300	1.0000 ± 0.1112	3.8084 ± 0.0491	**	4.2318 ± 0.1023	*	GAAACCCTACAAATGTGCTCAGTG	AGGTCTTCCCACAAGCAGTACAT	p < 0.01**
