# Supplementary material for: Co-expression analysis and identification of fecundity-related long non-coding RNAs in sheep ovaries
Source: Sci Rep. 2016 Dec 16;6:39398. doi: 10.1038/srep39398 (PMC5159859; doi:10.1038/srep39398)
Supplement: Supplementary Information [file srep39398-s1.pdf]

Supplementary Information

# Co-expression analysis and identification of fecundity-related long non-coding RNAs in sheep ovaries

Xiangyang Miao* Qingmiao Luo Huijing Zhao Xiaoyu Qin

Institute of Animal Sciences, Chinese Academy of Agricultural Sciences, Beijing, 100193, China

*Corresponding author: Xiangyang Miao Institute of Animal Sciences, Chinese Academy of Agricultural Sciences, Beijing, 100193, China, Tel: 86-10-62895663, Fax: 86-10-62895663 (China), E-mail: miaoxy32@163.com, mxy32@sohu.com

**Table S1** The identified genes and long non-coding RNAs in 16 modules

**Table S2** The enriched biological process for differentially expressed genes from HanBBvsDorset

**Table S3** The enriched biological process for differentially expressed genes from HanBBvsHan++

**Table S4** The enriched biological process for differentially expressed genes from Han++vsDorset

**Table S5** The enriched cellular component for differentially expressed genes from HanBBvsDorset

**Table S6** The enriched cellular component for differentially expressed genes from HanBBvsHan++

**Table S7** The enriched cellular component for differentially expressed genes from Han++vsDorset

**Table S8** The enriched molecular function for differentially expressed genes from HanBBvsDorset

**Table S9** The enriched molecular function for differentially expressed genes from HanBBvsHan++

**Table S10** The enriched molecular function for differentially expressed genes from Han++vsDorset

**Table S11** Cis-target genes of differentially expressed lncRNAs

**Table S12** Trans-target genes of partial differentially expressed lncRNAs

**Table S1** The identified genes and long non-coding RNAs in 16 modules

| colnames.datExpr0. | net.colors |
|---|---|
| TMEFF2 | yellow |
| LOC101119121 | blue |
| SLC3A1 | tan |
| LOC101119161 | green |
| TFEC | turquoise |
| TFF3 | lightcyan |
| HINT1 | yellow |
| ALS2CR12 | green |
| LOC101122204 | red |
| LOC101122205 | yellow |
| SCNN1B | red |
| SGPL1 | cyan |
| ACAP3 | brown |
| FUK | magenta |
| ZFHX2 | pink |
| LOC101120029 | cyan |
| FOS | brown |
| LOC101119221 | yellow |
| HK3 | purple |
| LOC101119256 | tan |
| LOC101119283 | greenyellow |
| ARAP2.1 | green |
| LOC101122107 | pink |
| SH2B2 | blue |
| CNTD1 | turquoise |
| GEN1 | salmon |
| XLOC_019586 | yellow |
| XLOC_019593 | turquoise |
| PTPN3 | turquoise |
| XLOC_005851 | green |
| GRN | black |
| MC1R | blue |
| F5 | purple |
| GK | red |
| DPP10 | cyan |
| LOC101122411 | yellow |
| OLR1 | green |
| IQUB | turquoise |
| XK | turquoise |
| EFHC2 | turquoise |
| PLA2G3 | salmon |

| | |
|---|---|
| TRNAW-CCA.19 | green |
| TRNAW-CCA.55 | blue |
| ADRB3 | tan |
| LOC101119013 | blue |
| HMGCS2 | blue |
| LOC101122301 | turquoise |
| CPT1B | red |
| LOC101122330 | purple |
| RAB3IP | magenta |
| ADRB2 | midnightblue |
| IRX4 | black |
| XLOC_029026 | cyan |
| XLOC_029019 | brown |
| DNASE1L2 | pink |
| PITX2 | cyan |
| LOC101122286 | green |
| LOC101114970 | greenyellow |
| RAB39B | yellow |
| LOC101114966 | blue |
| GAPT | green |
| USP36 | brown |
| USP35 | brown |
| USP43 | turquoise |
| LOC101122611 | black |
| CA4 | magenta |
| HS3ST2 | cyan |
| ISL2 | blue |
| XLOC_017093 | yellow |
| XLOC_042324 | pink |
| XLOC_019299 | salmon |
| C7H15orf57 | yellow |
| XLOC_005548 | turquoise |
| LOC100037673 | brown |
| HIRIP3 | midnightblue |
| TAC3 | pink |
| AQP4 | turquoise |
| CLEC4E | midnightblue |
| AKAP14 | turquoise |
| TMOD2 | greenyellow |
| TMOD1 | purple |
| CKM | brown |
| MFAP2.1 | yellow |
| HOXC9 | magenta |
| LRCOL1 | brown |

| | |
|---|---|
| HOXD3 | tan |
| GIPC2 | yellow |
| HOXB7 | brown |
| HOXB5 | midnightblue |
| CRH | blue |
| C17H12orf43 | midnightblue |
| XLOC_007390 | yellow |
| EFS | brown |
| P2RX5 | yellow |
| EBF3 | lightcyan |
| P2RX2 | red |
| CLDN7 | purple |
| GDF5 | yellow |
| VPS13C | purple |
| TPD52L1 | purple |
| TRNAE-UUC.29 | greenyellow |
| ARSE | purple |
| PPFIA3 | yellow |
| LOC101122803 | magenta |
| CBLN3 | red |
| XLOC_042153 | green |
| KIAA0825 | lightcyan |
| WDR72 | green |
| PCDH20 | tan |
| WDR54 | green |
| WDR52 | turquoise |
| WDR65 | turquoise |
| WDR63 | turquoise |
| WDR69 | turquoise |
| WDR38 | turquoise |
| WDR49 | turquoise |
| WDR46 | red |
| WDR16 | turquoise |
| XLOC_005122 | tan |
| LOC101122774 | salmon |
| WDR96 | turquoise |
| EHF | turquoise |
| KHDC3L | turquoise |
| ERF | blue |
| IGFBPL1 | salmon |
| FBXO15 | black |
| TRNAR-UCG.1 | salmon |
| PIWIL1 | greenyellow |
| PIWIL2 | brown |

| | |
|---|---|
| FHAD1 | turquoise |
| FAM65C | blue |
| FAM69C | red |
| XLOC_044102 | black |
| ARHGAP15 | black |
| XLOC_005052 | salmon |
| GNG3 | greenyellow |
| RHPN2 | green |
| GNB3 | magenta |
| ZBP1 | green |
| METTL21D | blue |
| PAH | blue |
| LOC101102772 | purple |
| ZBBX | turquoise |
| CREG1.1 | black |
| KRT7 | green |
| CENPI | turquoise |
| SSTR2 | turquoise |
| ACSBG1 | brown |
| LOC101109940 | green |
| OSBPL10 | brown |
| C3H2orf73 | turquoise |
| FAM8A1 | turquoise |
| ECT2L | turquoise |
| GPX2 | turquoise |
| DTHD1 | turquoise |
| RAB11FIP3 | brown |
| KIF21B | midnightblue |
| KIAA0430 | red |
| FAM83C | greenyellow |
| TIGD4 | black |
| TIGD5 | lightcyan |
| SYBU | lightcyan |
| KRT222 | midnightblue |
| REL | turquoise |
| LOC101102509 | magenta |
| ASIC4 | salmon |
| RAD51C | pink |
| SYT2 | greenyellow |
| BAI2 | midnightblue |
| BAI3 | magenta |
| LOC101102443 | turquoise |
| MLF1 | turquoise |
| XLOC_017148 | blue |

| | |
|---|---|
| MTSS1L | brown |
| KIAA0319 | cyan |
| BAIAP3 | turquoise |
| RNASEH2C | green |
| IGDCC3 | black |
| tRNA-Gln | blue |
| GJB1 | purple |
| RASGEF1A | turquoise |
| RALGAPA2 | green |
| LOC101107122 | salmon |
| OTOR | brown |
| FBXL19 | magenta |
| CSN1S1 | lightcyan |
| MREG | tan |
| SLC34A1 | red |
| PTGDR2 | greenyellow |
| KATNBL1 | purple |
| FAM92B | turquoise |
| ASPHD1 | blue |
| XLOC_029260 | green |
| LHB | turquoise |
| GILT | turquoise |
| LOC101107227 | purple |
| DPY19L2 | green |
| KATNAL2 | purple |
| RAB9B | midnightblue |
| UBXN10 | turquoise |
| PTGER4 | red |
| ANKFN1 | turquoise |
| CNTN4 | brown |
| RAB25 | turquoise |
| TMEM27 | blue |
| ATP2C2 | turquoise |
| RAB15 | green |
| XLOC_012276 | brown |
| SEPN1 | blue |
| PLA2G4B | magenta |
| MKX | red |
| MIA | blue |
| LOC101122022 | lightcyan |
| LOC101122021 | pink |
| MX2 | tan |
| ERP29 | lightcyan |
| TMEM82 | turquoise |

| | |
|---|---|
| TMEM8B | yellow |
| GM2A | pink |
| TMEM33 | blue |
| MPZ | blue |
| TMEM44 | green |
| FSTL5 | black |
| SORCS1 | greenyellow |
| LOC101102982 | pink |
| DGKB | blue |
| DGKQ | lightcyan |
| TSPAN1 | turquoise |
| TNFSF18 | black |
| NGB | purple |
| EID2 | greenyellow |
| ENKUR | turquoise |
| 5HT2A | turquoise |
| LOC101102907 | green |
| XLOC_019859 | brown |
| XLOC_027327 | yellow |
| AGBL4 | purple |
| XLOC_019813 | red |
| BEST1 | red |
| ORC6 | pink |
| LOC101107030 | salmon |
| SV2A | pink |
| ARHGEF38 | purple |
| PIBF1 | red |
| ARHGEF19 | black |
| ERICH2 | turquoise |
| ADRA1B | red |
| TRNAY-GUA.25 | brown |
| CPEB2 | green |
| DNALI1 | turquoise |
| SLC25A25 | red |
| SLC25A28 | purple |
| IRGQ.1 | brown |
| SLC25A37 | brown |
| LOC101106326 | cyan |
| TUBGCP6 | magenta |
| SLC25A40 | tan |
| ACRC | green |
| ADAMTS9 | pink |
| MUSK | green |
| CER1 | yellow |

| | |
|---|---|
| KIF9 | turquoise |
| NUDT13 | salmon |
| HS3ST3B1 | greenyellow |
| IL1RAPL1 | salmon |
| XLOC_015137 | pink |
| LOC101110755 | yellow |
| LOC101110773 | salmon |
| SPAG17 | turquoise |
| LOC101112900 | tan |
| TTC8 | turquoise |
| PPIL6 | tan |
| MUC1 | lightcyan |
| TRNAK-UUU.5 | tan |
| RINL | midnightblue |
| BST-2B | lightcyan |
| BST-2A | magenta |
| CHL1 | magenta |
| RAP1GAP | magenta |
| KLC2 | brown |
| PNMAL1 | midnightblue |
| PPP2R4 | turquoise |
| LOC101112920 | brown |
| C5H5orf46 | red |
| FAIM2 | greenyellow |
| LOC101112974 | green |
| LOC101110715 | black |
| LOC101110889 | greenyellow |
| KCNJ3 | yellow |
| KCNH1 | blue |
| MSR1 | magenta |
| ZO3 | green |
| TTC26 | red |
| TTC29 | turquoise |
| GM2A.1 | yellow |
| LOC101106059 | red |
| KCNC4 | blue |
| CHD5 | midnightblue |
| AMDHD2 | red |
| MYL10 | purple |
| TSPAN17.1 | brown |
| DIRAS2 | magenta |
| ROS1 | yellow |
| LOC101110150 | green |
| FAM154B | tan |

| | |
|---|---|
| LOC101102259 | yellow |
| XLOC_026611 | pink |
| ICOS | pink |
| MYO16 | blue |
| PRR22 | blue |
| GRIA2 | greenyellow |
| GRIA1 | greenyellow |
| KERA | black |
| LOC101106730 | yellow |
| FAM159A | brown |
| LOC101116002 | brown |
| LOC101110057 | red |
| POU2F3 | greenyellow |
| SYK | black |
| APOL6 | turquoise |
| FAM167B | red |
| XLOC_022217 | pink |
| ADAM33 | magenta |
| ADAM22 | black |
| MTNR1A | yellow |
| DNER | turquoise |
| PRRT2 | midnightblue |
| LOC101106658 | blue |
| LOC101116132 | green |
| ONECUT2 | purple |
| LOC101116109 | turquoise |
| SPDYA | turquoise |
| MED21 | yellow |
| FAM131A | midnightblue |
| C18H15orf26 | tan |
| ZMYND10 | turquoise |
| MYF5 | blue |
| TFAP2B | green |
| TPBG | cyan |
| LOC101102022 | blue |
| MED31 | black |
| LOC101106541 | turquoise |
| LOC101106588 | cyan |
| ITGAM | magenta |
| IFI6 | magenta |
| LOC101110481 | turquoise |
| LOC101102165 | red |
| CLGN | turquoise |
| SPG21 | pink |

| | |
|---|---|
| KANK3 | blue |
| NLRP14 | blue |
| GFRA4 | turquoise |
| GFRA3 | blue |
| LOC101106477 | salmon |
| LOC101106471 | purple |
| CHAC1 | blue |
| PODXL2 | black |
| VGF | blue |
| DPF1 | blue |
| XLOC_028961 | brown |
| XLOC_028954 | red |
| LOC101106418 | green |
| LOC101106434 | blue |
| B3GNT6 | brown |
| EFCAB1 | turquoise |
| PPP1R14D | black |
| PPP1R15A | black |
| FAM19A4 | salmon |
| IRF1.1 | turquoise |
| MPZL2 | tan |
| SH2D3A | turquoise |
| LOC101118745 | green |
| LOC101118734 | salmon |
| RBP2 | tan |
| FAM190A | turquoise |
| ZFP36 | black |
| FAM196A | green |
| MCOLN3 | pink |
| MCOLN2 | black |
| DNAI2 | purple |
| KRTAP1-1 | blue |
| DNAH7 | turquoise |
| DNAH5 | turquoise |
| DNAH6 | turquoise |
| LOC101114265 | brown |
| LOC101118607 | green |
| TRNAK-UUU.12 | purple |
| TNP2 | greenyellow |
| LOC101118660 | black |
| ALG1 | pink |
| LOC101114349 | turquoise |
| LOC101121777 | red |
| LOC101104869 | blue |

| | |
|---|---|
| LOC101104851 | magenta |
| PPP2R5B | tan |
| LOC101116228 | brown |
| LOC101116211 | yellow |
| PPP2R2B | turquoise |
| SERPINA6 | yellow |
| RPH3A | pink |
| LOC101121820 | magenta |
| LOC101118562 | lightcyan |
| C3H12orf5 | salmon |
| DSC2 | turquoise |
| CNIH2 | black |
| GATA1 | brown |
| LOC101116291 | blue |
| LOC101104731 | tan |
| CLIP2 | yellow |
| LOC101114011 | blue |
| LOC101116286 | turquoise |
| LOC101116281 | blue |
| LOC101104787 | black |
| AOAH | pink |
| SIRT4 | yellow |
| LOC101104797 | salmon |
| LSM11 | yellow |
| COX3 | midnightblue |
| TLL2 | midnightblue |
| BATF2 | lightcyan |
| TLE2 | brown |
| LOC101106844 | black |
| LOC101106847 | blue |
| CPA6 | blue |
| LOC101106881 | midnightblue |
| SIGLEC1 | brown |
| LOC101116302 | yellow |
| RARB | yellow |
| TRNAG-CCC.64 | tan |
| SUV420H2 | cyan |
| FAM183A | turquoise |
| FAM184B | red |
| ANKRD24 | brown |
| ANKRD35 | magenta |
| ANKRD37 | yellow |
| ANKRD45 | turquoise |
| LOC101114134 | blue |

| | |
|---|---|
| TLR6 | red |
| TLR8 | pink |
| ANKRD65 | turquoise |
| LAPTM5 | midnightblue |
| XLOC_036693 | salmon |
| FAM186B | greenyellow |
| LOC101114191 | brown |
| LOC101116846 | green |
| LOC101112305 | blue |
| LOC101104536 | black |
| CRIP3 | green |
| LOC101112251 | pink |
| LOC101116922 | black |
| LOC101118374 | pink |
| EPCAM | turquoise |
| LOC101116991 | green |
| C5H19orf21 | salmon |
| VGLL1 | green |
| CDC42EP2 | cyan |
| NPY5R | red |
| C5H19orf57 | blue |
| PERP | turquoise |
| LOC101112434 | yellow |
| RHOF | midnightblue |
| RARRES1 | purple |
| LOC101104431 | green |
| MUC13 | blue |
| C13H20orf96 | purple |
| RHCG | magenta |
| NUP62 | midnightblue |
| AGR2 | turquoise |
| LOC101104407 | black |
| AGMO | red |
| PECR | yellow |
| LOC101118267 | blue |
| LOC101118245 | green |
| AKD1 | turquoise |
| LOC101116650 | midnightblue |
| OXSR1.1 | greenyellow |
| C14H19orf18 | black |
| LOC101116694 | turquoise |
| XLOC_036962 | green |
| PHACTR3 | brown |
| MYH7B | brown |

| | |
|---|---|
| LOC101112487 | pink |
| LOC101118186 | red |
| LOC101112474 | pink |
| C20H6orf141 | salmon |
| XLOC_041882 | yellow |
| LOC101104348 | red |
| C-JUN.1 | brown |
| XLOC_004853 | green |
| LOC101116781 | black |
| MYH15 | yellow |
| PDE6B | brown |
| GDPD5 | cyan |
| GDPD3 | magenta |
| HOOK1 | turquoise |
| LOC101118060 | green |
| LOC101112593 | greenyellow |
| LOC101118086 | blue |
| S100A16 | blue |
| NYAP1 | brown |
| HECTD2 | turquoise |
| LOC101116801 | brown |
| NRXN2 | magenta |
| LOC101104286 | black |
| XLOC_041729 | brown |
| LOC101116828 | cyan |
| LOC101104264 | cyan |
| SKOR1 | blue |
| XLOC_043421 | brown |
| TRNAV-AAC.9 | purple |
| XLOC_041248 | purple |
| LOC101105609.1 | tan |
| TTC21A | green |
| LOC101121589 | green |
| PLEKHB1 | purple |
| TTC23L | purple |
| PLEKHD1 | green |
| LOC101123656 | pink |
| LOC101123657 | greenyellow |
| RPL39 | black |
| JUND | midnightblue |
| XLOC_018073 | blue |
| FCER1A | turquoise |
| IL5RA | turquoise |
| DOCK10 | red |

| | |
|---|---|
| LOC101121431 | green |
| VDAC1 | turquoise |
| BST1 | pink |
| LOC101123501 | yellow |
| ABCA9 | blue |
| XLOC_008930 | blue |
| NR4A1 | brown |
| CYB561 | green |
| LACE1 | tan |
| NEK10 | red |
| NEK11 | turquoise |
| CDCP2 | brown |
| XLOC_018337 | turquoise |
| PLXNB3 | tan |
| MRGPRF | magenta |
| PLXNC1 | magenta |
| COMMD6 | yellow |
| LY6G5B | cyan |
| SCN10A | brown |
| LOC101123401 | tan |
| LOC101123437 | purple |
| SLC6A20 | greenyellow |
| XLOC_041628 | blue |
| PLEKHS1 | turquoise |
| LOC101109655.1 | yellow |
| OSBPL7 | cyan |
| SLC46A3 | tan |
| RBM47 | purple |
| XLOC_004583 | green |
| MGST1 | pink |
| LOC101113094 | blue |
| LOC101115899 | green |
| EGFL8 | turquoise |
| NECAB3 | cyan |
| LOC101115876 | brown |
| PKIB | purple |
| C2H9orf135 | lightcyan |
| C2H9orf174 | turquoise |
| FBXL4 | pink |
| CAMKV | tan |
| PLB1 | pink |
| FOXJ1 | turquoise |
| ZGLP1 | green |
| DAGLA | red |

| | |
|---|---|
| IL23R | yellow |
| MAST1 | lightcyan |
| LOC101115749 | blue |
| SFTPA1 | blue |
| LOC101115768 | lightcyan |
| C3H12orf63 | turquoise |
| RNF32 | purple |
| CAMSAP3 | midnightblue |
| LOC101110014.1 | green |
| C21H11orf84 | cyan |
| XLOC_004234 | blue |
| C3H2orf50.1 | turquoise |
| AXDND1 | tan |
| PDZK1IP1 | green |
| SCG2 | brown |
| HTR1B | brown |
| HTR1F | green |
| NME9 | turquoise |
| NME5 | turquoise |
| XLOC_006360 | red |
| PNMT | brown |
| XLOC_004065 | cyan |
| MORN5 | turquoise |
| MORN2 | tan |
| PON2 | greenyellow |
| IL20RB | green |
| TRNAK-CUU.24 | salmon |
| NR0B1 | yellow |
| HDGF | brown |
| TACR1 | turquoise |
| SPOCK1 | black |
| ZNF784 | turquoise |
| MEGF6 | brown |
| PDZD7 | yellow |
| TMEM229B | yellow |
| LENG1 | brown |
| RIIAD1 | turquoise |
| LOC101103863 | green |
| CHRND | blue |
| NOD1 | salmon |
| STRA8 | salmon |
| HSF5 | green |
| TMPRSS5 | brown |
| BCORL1 | brown |

| | |
|---|---|
| LOC443167 | salmon |
| GPR52 | red |
| TMEM212 | purple |
| GPR21 | greenyellow |
| GPR18 | yellow |
| CAPSL | turquoise |
| CHRM3 | black |
| S100A1 | red |
| S100A2 | lightcyan |
| STRN4 | brown |
| CA5A | lightcyan |
| LOC101108781 | yellow |
| DGKE.1 | brown |
| MAPK15 | lightcyan |
| BCL2A1 | black |
| NOL4 | salmon |
| GBGT1 | brown |
| ZNF500 | green |
| ZNF529 | greenyellow |
| VWA3A | turquoise |
| VWA3B | turquoise |
| TCTE1 | green |
| MEIG1 | turquoise |
| KIAA1147 | yellow |
| PNPLA4 | yellow |
| MEIS3 | purple |
| C7H14orf166B | greenyellow |
| BFSP1 | magenta |
| XLOC_013348 | turquoise |
| NSUN7 | turquoise |
| HRH2 | midnightblue |
| LOC101108856 | blue |
| MATN3 | cyan |
| TMEM200C | magenta |
| LOC101101989 | turquoise |
| EMID1 | brown |
| RPS13 | pink |
| RTP1 | tan |
| CLDND2 | salmon |
| ZNF687 | brown |
| LOC443320 | yellow |
| LOC101108945 | turquoise |
| RPS3A | red |
| LOC101108140 | turquoise |

| | |
|---|---|
| LOC101108131 | greenyellow |
| LAMB3 | cyan |
| LRIG1.1 | black |
| STPG1 | lightcyan |
| CDKL1 | lightcyan |
| LOC101108102 | turquoise |
| DHX58 | magenta |
| LOC101123268 | greenyellow |
| NXPE2 | blue |
| KIAA1751 | turquoise |
| RNF182 | turquoise |
| LOC101123290 | brown |
| RNF175 | blue |
| ZNF366 | brown |
| ZNF300 | yellow |
| BICD1 | yellow |
| RNF166 | lightcyan |
| SPINT1 | red |
| NRGN | brown |
| RNF112 | magenta |
| METTL24 | turquoise |
| STX1B | greenyellow |
| NACAD | lightcyan |
| CDHR4 | turquoise |
| SPAG6 | turquoise |
| LOC101123128 | yellow |
| FMNL1 | purple |
| ZNF467 | red |
| SLC44A3 | salmon |
| SLC44A4 | turquoise |
| CDO1 | pink |
| DCDC2C | lightcyan |
| DCDC2B | turquoise |
| CDH1 | green |
| CDS1 | turquoise |
| FGF16 | midnightblue |
| C8H6orf165 | turquoise |
| RYR3 | brown |
| TMEM128 | pink |
| EMILIN3 | magenta |
| TMEM116 | pink |
| SLFN12L | green |
| LOC101123097 | yellow |
| FAM216B | turquoise |

| | |
|---|---|
| HILPDA | yellow |
| LOC101108423 | yellow |
| LOC101108480 | salmon |
| CRYBA4 | yellow |
| CAPS | turquoise |
| NLGN3 | greenyellow |
| ZNF182 | turquoise |
| CARS | cyan |
| ROPN1L | turquoise |
| CITED4 | blue |
| XLOC_018698 | purple |
| ZNF296 | yellow |
| PDE11A | lightcyan |
| TRIQK | red |
| LOC101108533 | brown |
| OSBP2 | brown |
| XLOC_043354 | tan |
| PKHD1L1 | blue |
| LOC101107441 | green |
| NFAT5.1 | green |
| LOC101105256 | yellow |
| LOC101109191 | black |
| IFFO1 | red |
| LRRN2 | brown |
| FAM221A | turquoise |
| SMIM8 | turquoise |
| LOC101109142 | red |
| SLC26A10 | magenta |
| FAM46B | magenta |
| CCDC85A | midnightblue |
| SLITRK6 | brown |
| NRBP2 | purple |
| REEP4 | yellow |
| KPNA7 | green |
| LOC101103164 | turquoise |
| FOXRED2 | green |
| LOC101102345.1 | pink |
| LOC101103153 | blue |
| LOC101103150 | yellow |
| WNT9B | black |
| CDC42BPA.2 | blue |
| PTP4A3 | black |
| CLN5.1 | blue |
| DHRS7 | yellow |

| | |
|---|---|
| LOC101109492 | tan |
| LOC101105437 | greenyellow |
| RSPH9 | turquoise |
| LOC101107613 | yellow |
| SNTN | turquoise |
| SP17 | turquoise |
| LOC101117063 | blue |
| LOC101103296 | pink |
| EME2 | brown |
| LOC101109338 | blue |
| ZDHHC7 | midnightblue |
| LOC101103216 | tan |
| LOC101105367 | black |
| LOC101107563 | cyan |
| AP1M2 | turquoise |
| SLC26A2.1 | red |
| LOC101111935 | greenyellow |
| LOC101111926 | purple |
| XLOC_015771 | brown |
| ENPP3 | turquoise |
| COL11A2 | tan |
| SCUBE1 | blue |
| ALDH8A1 | yellow |
| LOC101103317 | lightcyan |
| NFKBIA | greenyellow |
| CDR2L | brown |
| CCDC28B | magenta |
| XLOC_025521 | turquoise |
| MMP13 | yellow |
| ELN.1 | brown |
| XLOC_023265 | greenyellow |
| C1H1orf110 | green |
| KRT19 | magenta |
| KRT18 | purple |
| LOC101107832 | greenyellow |
| C1H1orf194 | purple |
| C1H1orf192 | brown |
| C1H1orf168 | turquoise |
| KIFC2 | magenta |
| BCL2L12 | lightcyan |
| LOC101103419 | turquoise |
| LOC101111712 | blue |
| LOC101111710 | pink |
| SYTL5 | cyan |

| | |
|---|---|
| TRMT61B | brown |
| LRRIQ3 | tan |
| LRRIQ1 | turquoise |
| CDRT4 | green |
| SLCO1C1 | greenyellow |
| WHSC2 | magenta |
| LOC101109564 | pink |
| LOC101117224 | turquoise |
| RSPH10B | turquoise |
| LOC101107743 | yellow |
| RPGRIP1L | turquoise |
| LOC101107776 | black |
| MARVELD1 | brown |
| FRMPD2 | green |
| LOC101105008 | salmon |
| LOC101103597 | turquoise |
| LOC101103570 | cyan |
| TRNAG-GCC.2 | blue |
| XLOC_025391 | blue |
| LOC101117365 | salmon |
| LOC101109865 | blue |
| LOC101111391 | blue |
| LOC101105090 | midnightblue |
| LOC101105099 | lightcyan |
| C24H16orf54 | turquoise |
| LOC101103624 | yellow |
| CCDC13 | turquoise |
| GRB7 | purple |
| CCDC37 | turquoise |
| CCDC39 | turquoise |
| CCDC136 | brown |
| ST14 | turquoise |
| TPPP3 | turquoise |
| CCDC160 | turquoise |
| SLC28A3 | turquoise |
| CCDC153 | turquoise |
| CCDC159 | cyan |
| CCDC78 | turquoise |
| RCAN2 | yellow |
| AKR1E2 | lightcyan |
| LOC101117455 | blue |
| INHBA.1 | yellow |
| ZFAND4 | tan |
| LOC101103749 | salmon |

| | |
|---|---|
| LOC101107996 | pink |
| PCBD1 | tan |
| LOC101119835 | brown |
| DUSP27 | tan |
| C23H18orf54 | turquoise |
| UROC1 | green |
| ICAM-1.1 | lightcyan |
| LOC101119869 | yellow |
| LOC101111150 | blue |
| SSBP4 | cyan |
| LOC101117587 | pink |
| HOXC13 | midnightblue |
| XLOC_032521 | blue |
| MYBPC2 | yellow |
| MYBPC1 | salmon |
| NETO1 | turquoise |
| XLOC_035213 | yellow |
| XLOC_020993 | tan |
| K2.12 | blue |
| CXHXorf22 | turquoise |
| LGI1 | salmon |
| PELP1 | brown |
| CXHXorf30 | turquoise |
| CXHXorf61 | black |
| DCDC2 | green |
| LOC101119773 | cyan |
| CCL11 | yellow |
| LOC101119765 | blue |
| DOCK2 | red |
| LOC101113545 | turquoise |
| CRISP3 | greenyellow |
| LOC101111310 | midnightblue |
| GSDMD | black |
| LOC101115488 | blue |
| LOC101111343 | blue |
| SHROOM1 | red |
| XLOC_045018 | turquoise |
| LOC101122944 | purple |
| LOC101122910 | blue |
| KIF1A | tan |
| XLOC_039472 | magenta |
| BBOX1 | blue |
| XLOC_039480 | cyan |
| LOC101113126 | greenyellow |

| | |
|---|---|
| GRXCR2 | blue |
| LOC101113134 | greenyellow |
| TRNAD-GUC.7 | cyan |
| XLOC_001041 | yellow |
| TRNAD-GUC.3 | black |
| PLIN1 | cyan |
| PHGDH | yellow |
| EMC1.1 | brown |
| LOC101122977 | blue |
| LRGUK | turquoise |
| PPP1R26 | magenta |
| L1CAM | brown |
| PPP1R32 | green |
| LOC101117804 | midnightblue |
| C15H11orf88 | green |
| C15H11orf71 | pink |
| LCN2 | pink |
| C15H11orf65 | purple |
| AFMID | yellow |
| DCAKD | brown |
| GYLTL1B | midnightblue |
| LOC101115687 | blue |
| LOC101113282 | red |
| LRRC23 | turquoise |
| LRRC36 | turquoise |
| LOC101113881 | brown |
| LRRC48 | turquoise |
| LRRC4B | black |
| LOC101117954 | greenyellow |
| TELO2 | brown |
| ZADH2.1 | salmon |
| WDFY4 | red |
| LOC101119456 | black |
| DNAJB13 | turquoise |
| LOC101119431 | blue |
| AGL | turquoise |
| LOC101105609 | brown |
| BLOC1S3 | brown |
| PACRG | turquoise |
| GABRA3 | magenta |
| AK5 | turquoise |
| AK8 | cyan |
| SHC3 | turquoise |
| SLC9A5 | magenta |

| | |
|---|---|
| LOC101120622 | blue |
| SLC17A5.1 | yellow |
| POLR3G | magenta |
| LAT2 | black |
| LOC101105919 | blue |
| LOC101113617 | green |
| XLOC_030735 | turquoise |
| LPAR3 | turquoise |
| LOC101109035 | salmon |
| LRRC10B | turquoise |
| NOVA2 | yellow |
| LOC101115106 | green |
| HOXD11 | salmon |
| LOC101113726 | cyan |
| TEKT3 | yellow |
| XLOC_001539 | tan |
| LOC101120476 | brown |
| LOC101119585 | blue |
| GCNT4 | lightcyan |
| LOC101115275 | midnightblue |
| KCTD17 | blue |
| KRIT1 | blue |
